# Assessing the Correlation of Periodontal Inflamed Surface Area (PISA) With Systemic Inflammatory Markers

**DOI:** 10.7759/cureus.62389

**Published:** 2024-06-14

**Authors:** Rosy Kumari, Anindita Banerjee, Abhishek Verma, Amrit Kumar, Nitubroto Biswas, Priyanka Kumari

**Affiliations:** 1 Department of Dentistry, Netaji Subhas Medical College and Hospital, Bihta, IND; 2 Department of Periodontology, Buddha Institute of Dental Sciences and Hospital, Patna, IND; 3 Department of Periodontology and Oral Implantology, Buddha Institute of Dental Sciences and Hospital, Patna, IND

**Keywords:** periodontitis, periodontal inflamed surface area (pisa), inflammatory markers, gingivitis, esr (erythrocyte sedimentation rate), c-reactive protein (crp) test

## Abstract

Background

Periodontitis has a vital role in eliciting a cross-reactivity or systemic inflammatory response, making periodontal inflamed surface area (PISA) a primary contributor to the inflammatory burden posed by periodontitis. PISA helps in the quantification of the amount of inflamed periodontal tissue. However, the existing literature data concerning PISA as an indicator of inflammatory burden are scarce, with limited research on the relationship between systemic inflammatory markers and PISA.

Aim

The present clinic-hematological cross-sectional study aimed to correlate PISA with systemic inflammatory markers. The study also aimed to assess serum concentrations of inflammatory markers such as erythrocyte sedimentation rates (ESR), C-reactive protein (CRP), and peripheral blood markers such as neutrophils and monocytes and to correlate these markers with PISA.

Methods

The study assessed 62 subjects, who were divided into two groups of 31 subjects, each following bleeding on probing (BOP) criteria. Group I consisted of subjects with generalized chronic gingivitis, and Group II included subjects with generalized chronic periodontitis. In two groups, BOP, probing pocket depth, clinical attachment level, and gingival recession were assessed along with PISA by a custom-made R function derived from a pre-existing, freely available MS Excel spreadsheet (Microsoft Corporation, Redmond, Washington). The results of the assessment were then compared.

Results

A statistically highly significant positive correlation was seen in PISA and CRP with a correlation coefficient of 0.4875 and p-value of 0.000059. A similar statistically significant positive correlation was seen in ESR and PISA with a correlation coefficient of 0.4089 and p-value of 0.000968. A statistically non-significant correlation was seen in neutrophils and PISA with p=0.576018. However, a moderate and positive statistically significant association was seen in monocyte and PISA with a correlation coefficient of 0.3258 and p-value of 0.009956.

Conclusions

The present study concludes that most of the common systemic inflammatory markers have a positive correlation with PISA. However, more studies are required to establish this correlation.

## Introduction

Periodontitis is a chronic inflammatory disease of the supporting tissues around the teeth. Severe periodontal diseases are reported to affect approximately 14% of the adult population worldwide, which represents more than one billion cases globally [1].

The Centers for Disease Control and Prevention (CDC) has published a recent report with data concerning the prevalence of periodontitis in adults in the U.S. (United States) and reported that 47.2% of adults aged 30 years or older have one or more forms of periodontal disease. Additionally, the CDC has reported that the prevalence of periodontitis among Indian adults was estimated to be approximately 62% between 2011 and 2020, with severe periodontitis affecting approximately 23.6% of the population. An increase in incidence is seen with the rise in age, with an incidence of 70.1% for periodontal disease in adults aged 65 years or older [2].

With periodontitis, the risk of progression and development of different diseases increases, including chronic kidney diseases, rheumatoid arthritis, arteriosclerosis, and type 2 diabetes mellitus [3]. The possible model for periodontitis development as a risk factor for other diseases can be attributed to the fact that periodontitis leads to an increase in inflammatory burden by increasing systemic inflammatory responses, eliciting bacteremia and cross-reactivity, which causes auto-immune reactions. This inflammatory reaction increases damage to the human body beyond the oral cavity. Following the plausible mechanism, it can be hypothesized that an increased quantity of inflamed periodontal tissue is associated with a higher likelihood of periodontitis eliciting cross-reactivity, systemic inflammatory responses, and bacteremia, which refers to the presence of bacteria in the bloodstream [4].

Following this assumption, a more recent parameter called PISA was developed to measure periodontitis as a risk factor for other diseases, which depicts the surface area of bleeding pocket epithelium in square millimeters (mm^2^) [5].

Gingivitis, which is usually the initial stage of periodontitis and is considered a site-specific inflammatory response, is developed from the accumulation of plaque biofilm and is characterized by edema and gingival redness with no periodontal attachment loss. The primary measure of gingivitis is bleeding on probing (BOP) scores and percentage, which is assessed as bleeding site proportion (yes/no) when stimulated with a standardized manual probe using controlled force to the bottom of the pocket or gingival sulcus at six sites, namely disto-lingual, lingual, mesio-lingual, disto-buccal, buccal, and mesiobuccal, on all the teeth present [6].

BOP is an indicator of a reduction in epithelial integrity and thickness, increased blood vessel fragility and density, and decreased collagen density. Discontinuous, fragile, and thin pocket epithelium can serve as an entrance for oral bacteria to the systemic circulation. Additionally, BOP has the characteristic feature of dense infiltration or inflammatory cells. These inflammatory cells have a vital role in eliciting a cross-reactivity or systemic inflammatory response. Hence, PISA can be considered a primary contributor to the inflammatory burden posed by periodontitis [7].

It has been reported in the literature that inflammation increases systemic inflammatory markers such as monocytes, neutrophils, ESR, and CRP. PISA helps in the quantification of the amount of inflamed periodontal tissue. However, the existing literature data concerning PISA as an indicator of inflammatory burden are scarce, with limited research on the relationship between systemic inflammatory markers and PISA [8].

Hence, the present study aimed to correlate systemic inflammatory markers and PISA in subjects suffering from chronic generalized periodontal diseases. Therefore, the present study aimed to correlate PISA with systemic inflammatory markers. The study also aimed to assess serum concentrations of inflammatory markers such as ESR, CRP, and peripheral blood markers such as neutrophils and monocytes and to correlate these markers with PISA.

## Materials and methods

The present clinic-hematological cross-sectional study aimed to correlate periodontal inflamed surface area (PISA) with systemic inflammatory markers. The study also aimed to assess serum concentrations of inflammatory markers such as ESR, CRP, and peripheral blood markers such as neutrophils and monocytes and to correlate these markers with PISA. The study was conducted at the Buddha Institute of Dental Sciences & Hospital, Patna, Bihar, India. The study subjects were from the Department of Periodontology of the Institute. We have received clearance from the concerned Institutional Ethical Committee, Buddha Institute of Dental Sciences & Hospital, Patna, Bihar, India (IEC/144/BIDSH/2020). The study period was 18 months, which started in January 2021 and ended in June 2023. The purpose and design of the study were verbally explained to each patient, and written informed consent was obtained from each of them.

The study assessed 62 subjects, and the sample size was determined using the estimated values from the literature using the formula: Total sample size = N = ((Zα+Zβ)/C)2 + 3, where Zα is the z variate of the alpha error, i.e., a constant with value 1.96; Zβ is the z variate of beta error, i.e., a constant with value 0.84; and C = 0.5 ln((1+r)/(1-r)). Approximate estimates were 80% power, Type I error of 5%, Type II error of 20%, and a minimum correlation between the two techniques of 0.30. A minimum of 62 samples needed to be taken in this present study.

The inclusion criteria for the study were subjects that were systemically healthy as assessed from the history of the subject, subjects having more than 20 teeth, excluding the third molar, and subjects that were willing to participate in the study. The exclusion criteria for the study were subjects that required any systemic medications such as non-steroidal anti-inflammatory drugs (NSAIDs) such as ibuprofen, aspirin, and naproxen sodium within the past six months, obese subjects having a body mass index (BMI) of ≥ 30 kg/m^2^, subjects that used tobacco in any form, chronic alcoholics, pregnant or lactating females, subjects with gingival overgrowth or pseudo pockets, and subjects that did not give consent for study participation.

The study assessed 62 subjects that fulfilled the inclusion criteria, and these subjects were divided into two groups of 31 subjects each following BOP criteria by Armitage in 1990 [7]. Group I was comprised of subjects with generalized chronic gingivitis, and Group II included subjects with generalized chronic periodontitis. After final inclusion, the subjects were given a pre-procedural mouth rinse using 0.2% of 10 mL chlorhexidine for one minute. This was followed by a clinical history recording with complete medical and dental history.

A comprehensive periodontal examination was done to assess periodontal parameters such as BOP, probing pocket depth, clinical attachment level, and gingival recession using a generation pressure-sensitive periodontal probe with a pressure of 20 g and UNC-15 (GDC, Hoshiarpur, India) markings with color coding at 5, 10, and 15 mm. This was placed parallel to the long axis of the tooth at each site to assess all periodontal parameters at the six sites, namely, mesiobuccal, mid-buccal, distobuccal, mesio-lingual, mid-lingual, and disto-lingual. A single operator expert in the field recorded all the parameters. BOP was assessed after 30 seconds of probing at all three sites at the buccal, lingual, and palatal surfaces of each tooth. For the presence or absence of BOP, a dichotomous scoring system was used, recorded as Yes or No.

PISA was assessed using the average surface area of each tooth type along with periodontal measures using a custom-made R function derived from a pre-existing, freely available MS Excel spreadsheet (Microsoft Corporation, Redmond, Washington) (https://www.parsprototo.info) as recommended by Nesse et al. in 2008 [5].

PISA was assessed in four steps using an Excel spreadsheet. After filling periodontal probing depth (PPD) measurements at six sites per tooth, the computer calculated the mean PPD for each particular tooth. The mean PPD value around a particular tooth was entered into a formula that translated this linear mean PPD into a periodontal epithelial surface area (PESA) for that specific tooth. The PESA for a particular tooth was the root surface area of that tooth (in mm), i.e., covered by a pocket epithelium. The mean probing pocket depth around a particular tooth was entered into a formula that translated this linear mean PPD into a PESA for that specific tooth. The PESA for a particular tooth was the root surface area of that tooth (in mm^2^), i.e., covered by a pocket epithelium. PISA may also comprise an uninflamed pocket epithelium that does not pose an inflammatory burden. Therefore, for a particular tooth, it is subsequently multiplied by the proportion of sites around that tooth that were affected by BOP. For example, if three out of six sites were affected by BOP, the PESA of that particular tooth was multiplied by 3/6, and this gives the PISA of that specific tooth. The sum of PISAs around each tooth was calculated, and this resulted in the total PISA of a patient.

A 5 mL venous blood was collected following aseptic and sterile conditions and was sent to the Department of Oral Pathology and Microbiology of the same institute for the evaluation of the CRP, ESR, monocyte, and neutrophil counts. All the investigations were done in a single laboratory.

Data were analyzed statistically using IBM SPSS Statistics for Windows, Version 26 (Released 2019; IBM Corp., Armonk, New York) and were expressed as means and standard deviations. The normality of numerical data was checked using the Shapiro-Wilk test. The non-parametric tests used were the Mann-Whitney U test for intergroup comparisons and Spearman’s rank correlation test for bivariate correlation between two numerical variables. For all statistical analyses, p<0.05 was considered to be statistically significant, ensuring an α error of 5% and a β error of 20%. This provided the study with a statistical power of 80%.

## Results

The present study comprised 62 subjects, which were divided into two groups, i.e., Group I: patients with generalized chronic gingivitis and Group II: patients with generalized chronic periodontitis. PISA was calculated using a programmed Microsoft Excel spreadsheet. In every patient, peripheral blood was collected to estimate the CRP, ESR, neutrophil count, and monocyte count. Regarding the comparison between Group I and Group II based on PISA values, the mean PISA value was compared within the two groups by using the Mann-Whitney U test. The mean PISA value in Group I was 868.96±75.71 mm^2^ and 1704.68±287.01 mm^2^ in Group II. The difference was highly significant between the groups, with p<0.01 for PISA (mm^2^), indicating that Group II had greater values (periodontitis), as shown in Table 1.

**Table 1:** Comparison between Group I and Group II based on PISA values **Correlation is significant at the 0.01 level (two-tailed) N: number of subjects

Group	N	Mean (mm^2^)	Standard Deviation	Standard Error Mean	Z-value	Mann-Whitney U Test p-value
I	31	868.96	75.71	13.60	-6.758	0.000**
II	31	1704.68	287.01	51.55		

The study results showed a comparison between Group I and Group II concerning different inflammatory markers using the Mann-Whitney U test. The mean CRP value in Group I was 1.25±1.24 mg/L and 2.32±1.05 mg/L in Group II, showing a statistically highly significant difference in the values between the groups (p<0.01) for CRP (mg/L) with higher values in Group II. For ESR, the mean ESR value in Group I was 7.08±1.94 mm and 8.65±3.77 mm in Group II, depicting a statistically non-significant difference with p=0.1443. On comparing neutrophil counts, the mean count (n×1000/μL) in Group I was 4.16±1.43 and 4.14±1.14 in Group II, showing a statistically non-significant difference with p=0.67448. For comparison between Group I and Group II concerning monocyte count, the mean monocyte count (n×1000/μL) in Group I was 0.27±0.07 and 0.31±0.07 in Group II, depicting a statistically non-significant difference with p=0.1443 (Table 2).

**Table 2:** Comparison between Group I and Group II concerning different inflammatory markers **Correlation is highly significant (p<0.01, two-tailed) ^#^Statistically non-significant (p>0.05) CRP: C-reactive protein; ESR: erythrocyte sedimentation rates; N: number of subjects

Inflammatory Markers	Group	Mean (mm^2^)	Standard Deviation	Standard Error Mean	Mann-Whitney U value	Z-value	p-value	
CRP (mg/L)	I	1.25	1.24	0.22	206	-3.857	0.00012**	
	II	2.32	1.05	0.19				
ESR (mm)	I	7.08	1.94	0.35	376.5	-1.457	0.1443^#^	
	II	8.65	3.77	0.68				
Neutrophil (n×1000/μL)	I	4.16	1.43	0.26	450.5	-0.415	0.67448^#^	
	II	4.14	1.14	0.20				
Monocyte (n×1000/μL)	I	0.27	0.07	0.01	342.5	-0.415	0.1443^#^	
	II	0.31	0.07	0.01				

It was seen that on assessing the correlation between different inflammatory markers and PISA values for all subjects (Group I + Group II), a statistically highly significant positive correlation was seen between PISA and CRP with a correlation coefficient of 0.4875 and a p-value of 0.000059. A similar statistically significant positive correlation was seen between ESR and PISA with a correlation coefficient of 0.4089 and a p-value of 0.000968. A statistically non-significant correlation was seen between neutrophils and PISA with p=0.576018. However, a moderate and positive statistically significant association was seen between monocytes and PISA with a correlation coefficient of 0.3258 and a p-value of 0.009956 (Table 3).

**Table 3:** Correlation between different inflammatory markers and PISA values for all subjects (Group I + Group II) **Correlation is highly significant (p<0.01, two-tailed) CRP: C-reactive protein; ESR: erythrocyte sedimentation rates; N: number of subjects

Parameter	CRP (mg/L)	ESR (mm)	Neutrophil (n×1000/μL)	Monocyte (n×1000/μL)
Correlation coefficient	0.4875**	0.4089**	0.0724	0.3258**
p-value	0.000059	0.000968	0.576018	0.009956
N	62	62	62	62

For the intragroup comparison of correlation between different inflammatory markers and PISA within Group I (gingivitis), a statistically non-significant correlation was observed between PISA and CRP, ESR, neutrophils, and monocytes with respective correlation coefficients of 0.1439 mg/L, 0.147 mm, -0.0017 n×1000/µL, and 0.3416 n×1000/µL, respectively, and respective p-values of 0.439931, 0.430037, 0.99574, and 0.059997, as summarized in Table 4.

**Table 4:** Correlation between different inflammatory markers and PISA within Group I (gingivitis) ^#^Statistically non-significant (p>0.05) PISA: periodontal inflammatory surface area; CRP: C-reactive protein; ESR: erythrocyte sedimentation rates; N: number of subjects

PISA (mm^2^)		CRP (mg/L)	ESR (mm)	Neutrophil (n×1000/μL)	Monocyte (n×1000/μL)
	Correlation coefficient	0.1439^#^	0.147^#^	-0.0017^#^	0.3416^#^
	p-value	0.439931	0.430037	0.99574	0.059997
	N	31	31	31	31

It was seen that on intragroup comparison of correlation between different inflammatory markers and PISA within Group II (periodontitis), a statistically significant and positive correlation was seen between PISA and CRP and PISA and ESR with respective correlation coefficients of 0.3843 mg/L and 0.468 mm, and p-values of 0.032802 and 0.00793, respectively. However, a statistically weak correlation was seen between PISA and neutrophils and PISA and monocytes with respective correlation coefficients of 0.2907 n×1000/µL and 0.2366 n×1000/µL, respectively, and p-values of 0.112622 and 0.2012, respectively, as shown in Table 5.

**Table 5:** Correlation between different inflammatory markers and PISA within Group II (periodontitis) *Correlation is significant at the 0.05 level (two-tailed) **Correlation is significant at the 0.01 level (two-tailed) ^#^Statistically non-significant (p>0.05) CRP: C-reactive protein; ESR: erythrocyte sedimentation rates; N: number of subjects

PISA (mm^2^)		CRP (mg/L)	ESR (mm)	Neutrophil (n×1000/μL)	Monocyte (n×1000/μL)
	Correlation coefficient	0.3843*	0.468**	0.2907^#^	0.2366^#^
	p-value	0.032802	0.00793	0.112622	0.2012
	N	31	31	31	31

## Discussion

According to the latest data, periodontitis was estimated to occur in approximately 62% and severe periodontitis in 23.6% of Indian adults between 2011 and 2020. To assess the severity of the periodontal diseases, BOP, PPD, and CAL are clinically evaluated, and bone loss is assessed radiographically. These parameters are specific to each tooth, with no cumulative values that assess the total inflammatory status of the periodontium. Hence, a PISA was developed that considers BOP and PPD to evaluate the surface area of the bleeding pocket epithelium. This area represents the active inflammatory status imparted by the periodontium on systemic tissues, as suggested by Nesse et al. in 2008 [5]. The wide application of PISA can be conclusive on periodontitis as a risk factor for systemic diseases. Various literature studies have reported a link between poor oral health and cardiovascular diseases, type 2 diabetes, adverse pregnancy outcomes, osteoporosis, aspiration pneumonia, and rheumatoid arthritis. However, various prospective studies are needed to assess if these diseases are linked simply because they have risk factors in common or if a true causal relationship exists, as reported by Cullinan et al. in 2009 [8].

The study results showed that the mean PISA was compared within two groups using the Mann-Whitney U test. The mean PISA value in Group I was 868.96±75.71 mm^2^ and 1704.68±287.01 mm^2^ in Group II. The difference was highly significant between the groups, with p<0.01 for PISA (mm^2^) with higher values in Group II (periodontitis). These results were consistent with the study of Park et al. in 2017 [9], where the authors concluded that an increased PISA value was associated with increased periodontal disease severity. Other studies by Leira et al. in 2018 [10] and Anil et al. in 2021 [11] also reported similar results in periodontal subjects that were systemically healthy, where the mean PISA value was lowest and highest in periodontally healthy and severe periodontitis groups, respectively. The PISA difference between groups was highly significant in these studies as well as in the present study.

An association has been established between CRP and PISA, with CRP being considered a key biomarker of systemic inflammation, as suggested by Bansal et al. in 2014 [12]. A significant increase in CRP was seen in subjects with severe periodontitis compared to healthy subjects, as reported by Miki et al. in 2021 [3] and Slade et al. in 2003 [13]. It can be attributed to the fact that inflammatory mediators such as TNF-α, IL-6, and IL-1 are released during periodontitis, which stimulates hepatocytes to produce CRP, as reported by Bansal et al. [12]. Additionally, studies by Park et al. in 2017 [9] and Kalburgi et al. in 2014 [4] confirmed the association between CRP levels and PISA in systemically healthy subjects, similar to the results of the present study depicting significantly higher CRP in Group II compared to Group I subjects. Also, the study by D' Aiuto et al. in 2004 [14] concluded that CRP levels decreased with the control of periodontitis.

Another study by Miki et al. [3] hypothesized that in the future, CRP might be predicted in subjects with PISA≥500 mm^2^ without blood sampling. Additionally, it can be easier for internists and dentists to work together by utilizing PISA as a common term for assessing the risk of systemic diseases from periodontitis. If future research establishes PISA as a marker for CRP, it will bridge medicine and dentistry and pose great social significance.

ESR is another inflammatory marker and is less specific and sensitive to inflammation than CRP. However, various studies depicted that subjects with chronic periodontitis have higher levels of ESR compared to healthy subjects, as reported by Kalsi et al. in 2017 [15] and Merchant in 2002 [16], and a significant decrease is reported after periodontal therapy. The results of the present study show an increase in PISA value with increased ESR in subjects with periodontitis. This is vital considering that ESR change is vital in low-grade joint and bone infections, as also mentioned in the study by Harrison in 2015 [17]. Also, periodontitis is a low-grade infection that affects and destroys the alveolar bone at a low pace. The correlation could not be established for PISA and ESR in gingivitis subjects as gingivitis does not include the alveolar bone.

Carneiro et al. in 2012 [18] reported that in periodontitis, the primary defense against bacterial challenge is phagocytosis by monocytes and neutrophils, and their concentration is also vital as systemic inflammatory markers. The study results showed a non-significant decrease in neutrophils and an increase in monocytes from gingivitis to periodontitis. This can be attributed to the fact that neutrophils dominate in early periodontal lesions characterizing gingivitis. However, neutrophil concentration decreases from the shift of gingivitis to periodontitis, whereas lymphocytes and plasma cells predominate, as Kaur et al. documented in 2021 [19]. The existing literature data considering this are controversial and scarce, reporting both the increase and reduction of phagocytosis by neutrophils, as also suggested by Carneiro et al. in 2012 [18].

No other existing literature study reported a correlation of monocytes and neutrophils with PISA in subjects with chronic generalized gingivitis and generalized chronic periodontitis. The present study reported a strong correlation between monocytes and PISA and a weak relationship between neutrophils and PISA.

Limitations

The present study holds various limitations, including the fact that PISA cannot accurately quantify the inflamed periodontal tissues in the subjects with gingival overgrowth and pseudo pockets, and such subjects were excluded from the study. The study included only systemically healthy subjects. However, no biochemical assessment was done to ensure systemic health. The study assessed only the dental and medical history provided by the subjects, including inquiring about common disease symptoms. A detailed history was recorded, although it is not entirely reliable in confirming that subjects are free of diseases that could lead to an increase in inflammatory markers. Inflammatory markers are also affected by pregnancy, obesity, smoking, and age. However, pregnant females and obese subjects were excluded, and age was not considered. The systemic inflammatory markers in patients not suffering from any periodontal diseases have not been studied. Additionally, the study had a small sample size.

## Conclusions

PISA is positively correlated with the majority of popular systemic inflammatory indicators. The PISA value increases with the severity of periodontal disease. There was a considerable PISA difference between periodontitis and gingivitis. When comparing people with severe PISA to healthy participants, a substantial increase in CRP was seen. In participants with periodontitis, a higher ESR was associated with an increase in PISA score. We discovered a strong link between monocytes and PISA and a non-significant relationship between neutrophils and PISA.

In order to prove this association, additional research is necessary. Patients with chronic generalized gingivitis had a lower PISA value than those with generalized chronic periodontitis, and this difference is statistically significant. PISA increases are accompanied by increases in CRP, ESR, neutrophils, and monocytes.
